# The protective role of steroids on coronary arteries in the acute phase of Kawasaki disease for high risk patients: a retrospective study

**DOI:** 10.3389/fped.2025.1636339

**Published:** 2025-08-28

**Authors:** Alessandra Marchesi, Rosanna Recupero, Letizia Sardella, Riccardo Babini, Livia Gargiullo, Isabella Tarissi de Jacobis, Nicola Cotugno, Elena Bozzola, Maria Rosaria Marchili, Fabrizio De Benedetti, Alberto Villani

**Affiliations:** ^1^ Department of General Pediatrics and II Level Emergency, IRCCS Bambino Gesù Children’s Hospital, Rome, Italy; ^2^ Department of Pediatrics, A.O.U. San Giovanni di Dio e Ruggi D'Aragona, Salerno, Italy; ^3^ Pediatrics Residency, University of Insubria, Filippo del Ponte Hospital, Varese, Italy; ^4^ Consultant, Bernareggio, Italy; ^5^ Department of Systems Medicine, University of Rome “Tor Vergata”, Rome, Italy; ^6^ Clinical Immunology and Vaccinology Unit, IRCCS Bambino Gesù Children’s Hospital, Rome, Italy; ^7^ Rheumatology Unit, IRCCS Bambino Gesù Children's Hospital, Rome, Italy

**Keywords:** Kawasaki disease, steroids, coronary arteries aneurysm, high risk patients, coronary arteries aneurysms, IVIG resistance

## Abstract

**Purpose:**

The incidence of coronary artery aneurysms in Kawasaki disease has been increasing for reasons that remain unclear. Recently the use of corticosteroids in KD has been reconsidered for high-risk patients. To evaluate their effectiveness as first line therapy in combination with IVIG and aspirin for selected KD patients, we performed this retrospective single-center study.

**Methods:**

We retrospectively analyzed KD patients (aged 30 days to 18 years) who were hospitalized at the Bambino Gesù Children's Hospital between December 2007 and February 2023. The patients were classified as “high risk (HR)” or “low risk (LR)” for IVIG resistance and/or developing CAA according to the Italian guidelines and were stratified by treatment. We also analyzed changes in AHA risk class in the HR-S and HR-NS groups. Furthermore, to obtain a single representative Z-score for each patient, we introduced a novel calculation method: a. Z-MAX: the maximum value of the scores between the three coronary arteries. b. Z-SUM: the sum of the single Z-scores of the three coronary arteries.

**Results:**

374 patients were enrolled, 78 with CAA and 296 without CAA. Of those, 195 patients were classified as HR and all subsequent analyses in this study were exclusively conducted on this subgroup. At the end of the eight weeks, the HR-S group showed a significant reduction in coronary artery dimensions expressed as Z-score (reduction of 65% in RCA, 63% in LMCA, and 36.5% in LAD). In contrast, the HR- NS group showed an increase in the dimension of two arteries (RCA increased by 17% and LAD increased by 15%) and a slight reduction of LMCA (7.5%).

**Conclusion:**

First-line therapy with IVIG plus steroids in HR-KD patients reduces the development of CAA.

## What is Known—What is New

It is well established that preventing CAA in KD is a key goal in reducing long-term cardiovascular morbidity. Although early treatment with IVIG reduces their occurrence, the prevalence of IVIG resistance and the risk of CAA have increased in recent decades. Our study showed that early adjunctive steroid therapy—a safe, manageable, and cost- effective approach—significantly reduces the development of CAA in selected high-risk patients.

## Introduction

Kawasaki disease (KD) is an acute multisystemic inflammatory vasculitis that primarily affects medium-sized arteries, particularly the coronary arteries (1, 2). It occurs most frequently in children under five years of age (2). The main complications are arterial aneurysmal dilatations, particularly coronary artery aneurysms (CAA), especially in untreated cases. Preventing these complications is a key objective in reducing long-term cardiovascular morbidity.

Coronary artery dilatation is currently classified according to American Heart Association (AHA) guidelines (2) as follows:

• Mild ectasia: Z-score 2–2·5 SD
• Small aneurysm: Z-score 2·5–5 SD
• Medium aneurysm: Z-score 5–10 SD
• Giant aneurysm: Z-score ≥ 10 SD (3).

Most CAAs involve the proximal segments; isolated distal involvement is rare. The left coronary artery, especially the left anterior descending artery (LAD), is the most frequently affected. Approximately 30% of patients present with multiple CAAs (4).

Randomized controlled trials and meta-analyses have clearly demonstrated that timely administration of intravenous immunoglobulin (IVIG) reduces the incidence of CAA (5), lowering it from about 20% in untreated cases to 4% in treated ones (6).

However, IVIG resistance occurs in up to 20%–40% of patients and is associated with an increased risk of CAAs (7, 8). Several studies have reported persistently high rates of coronary complications despite timely IVIG treatment (9), for reasons that remain poorly understood (10).

Corticosteroids are commonly used to treat other forms of vasculitis, but have seen limited application in KD due to unclear etiological mechanisms. However, they could be effective due to their potent anti-inflammatory and immunosuppressive effects.

Historically, the first physician to use steroids for acute KD was Dr Tomisaku Kawasaki himself. He treated 21 patients with various forms of parenteral or oral steroids, but their use was initially avoided for the treatment of KD based on the findings of Kato's (11) 1979 report that concluded an increased risk of cardiovascular complications. He described that, one to two months after the onset of the disease, coronary angiography demonstrated coronary aneurysms in 64.7% of cases in the group treated with monotherapy using steroids. In view of the fact that thrombotic occlusion of a coronary artery was the direct cause of sudden death due to KD, aspirin was preferred as an effective means of preventing sudden death.

Since IVIG was elected as the gold standard therapy for KD first-line treatment², later studies reported the results of combined steroid and IVIG therapy (12–14).

Considering the possible etiopathogenic mechanism during the acute phase of KD, monocytes play a central role among the inflammatory cells and are a major source of the cytokine storm, particularly in patients with CAAs.

Steroids are known to downregulate inflammatory mediators, limit leukocyte migration, and reduce capillary permeability.

A small, preliminary study published in 2024 systematically evaluated the molecular mechanisms of combined IVIG and methylprednisolone therapy in KD patients. It showed that IVIG plus methylprednisolone suppresses more monocyte-mediated inflammatory responses than IVIG alone, enhances NK cell cytotoxicity and inhibits B cell activation than IVIG alone and involve multiple ways of the inflammatory response process, resulting in a more pronounced anti-inflammatory effect. These effects occurred through: increasing the number of lymphocytes (e.g., CD4+ T and CD8+ T cells), decreasing the number of inflammatory cells (e.g., monocytes), inhibiting B cell activation, upregulating the expression of interferon-related genes, in CD4+ T cells, CD8+ T cells, and B cells, enhancing NK cell cytotoxicity by regulating receptor homeostasis, significantly reducing the expression level of pro-inflammatory cytokines (e.g., CXCL5, CCL19, IL17C, et al.), which IVIG treatment alone failed to significantly reduce (15).

Historically, challenges in adopting corticosteroid therapy stemmed from heterogeneity in dosing regimens and study populations, as seen in early trials by the American Pediatric Heart Network (12), the Japanese “RAISE” study (13) as well as the large post-RAISE observational study (14). A subsequent meta-analysis of 16 comparative studies (including both observational and randomized trials) involving 2,746 KD patients showed that the early addition of steroids to IVIG significantly reduced the risk of CAAs (odds ratio 0·424; 95% CI, 0·270–0·665) (16). Multiple clinical studies have also confirmed the safety of corticosteroid use in severe KD (14, 17).

Despite these findings, the question of whether to add corticosteroids to first-line KD therapy remains unresolved.

Another area of contention involves risk stratification scores, which were originally developed for Eastern populations and demonstrate poor sensitivity in Caucasian patients (18), prompting further efforts to identify high-risk patients. Eleftheriou et al. (19) identified risk factors including age ≤ 12 months, elevated C-reactive protein (CRP), severe anemia, hypoalbuminemia, liver disease, macrophage activation syndrome, KD shock syndrome, and coronary aneurysms at disease onset. Son et al. (20) later identified additional predictors of coronary aneurysm development in North American populations within two to eight weeks: CA Z-score >2 on the initial echocardiogram, age <6 months, Asian ethnicity, and CRP ≥ 13 mg/dl.

Based on these data, several clinical protocols and guidelines have been proposed, including the 2018 Italian Society of Pediatrics Guidelines (3), and the 2021 American College of Rheumatology/Vasculitis Foundation Guidelines (21) and AHA 2024 Guidelines (22), all of which recommended IVIG plus corticosterids as first-line therapy in high- risk patients to prevent the progression of CAA.

However, the 2021 American Guidelines define neither the optimal steroid dosage nor duration, highlighting the need for further research in Western populations (21).

The Italian Guidelines provide a stratified approach, classifying patients as low-risk (LR) or high-risk (HR) for CAA development and/or IVIG non-response (3). Treatment protocols include: in low-risk patients (LR):

- First line therapy: IVIG 2 g/kg
- Second-line treatment for non-responders:
  ○ second infusion of IVIG 2 g/kg
  ○ in case of persisting absence of response to treatment: methylprednisolone (bolus 30 mg/kg/day) for three consecutive days, followed by prednisone (oral 2 mg/kg/day, gradually tapered according to inflammatory markers)

in high-risk patients (HR):

- First line therapy: IVIG 2 g/kg plus methylprednisolone (single bolus 30 mg/kg)
- Second-line treatment for non-responder patients: second infusion of IVIG 2 g/kg plus methylprednisolone (i.v. 30 mg/kg/day, for three consecutive days), followed by prednisone (see above).

The 2024 AHA scientific statement further supports intensified initial therapy in high-risk KD, recommending steroids, infliximab, or etanercept (22).

To observe the efficacy of first-line IVIG plus steroids in HR KD patients, we conducted a retrospective single-center cohort study with three times follow- ups, assessing:

1. reduction in CAA size according to Z-score at 2, 4, 8-week follow-ups;
2. changes in AHA cardiovascular risk class.

The choice and duration of antiplatelet or anticoagulant therapy were not evaluated in this study.

## Materials and methods

This study includes KD patients aged 30 days and 18 years who were admitted at Bambino Gesù Children's Hospital, from December 2007 to February 2023.

Diagnosis followed the AHA guidelines (2), and the study was complied with Good Clinical Practice and the principles of the Declaration of Helsinki for ethical research.

Patients were stratified as high risk (HR) or low risk (LR) using the Italian Guidelines (3). We defined high-risk patients as individuals who met one or more of these specified criteria:

- age ≤ 12 months
- C-reactive protein > 200 mg/L
- severe anemia (Hb < 8 g/dl)
- albumin level < 2·5 g/dl
- liver involvement (ALT > 100 mg/dl, AST > 200 mg/dl)
- coronary artery aneurysms (Z- score > 2)
- macrophage activation syndrome
- Kawasaki disease shock syndrome (KDSS).

Four treatment groups were defined:

• HR-S: High-risk patients treated with IVIG and steroids as first-line therapy
• HR-NS: High-risk patients who were only treated with IVIG and never with steroids as a first-line therapy
• LR-S: Low-risk patients who were treated with IVIG and steroids as a first-line therapy
• LR-NS: Low-risk patients who only received IVIG and were never treated with steroids as a first-line therapy.

The treatment regimens followed the KD Italian Guidelines (3), i.e., methylprednisolone (i.v. bolus of 30 mg/kg), followed by prednisone (os of 2 mg/kg/day, which was gradually tapered according to inflammatory markers).

The clinical, laboratory and echocardiography data of the patients were collected in a database using an Excel spreadsheet^Ⓡ^ and subsequently processed.

Echocardiographic assessments were conducted by the paediatric cardiology team at Bambino Gesù Children's Hospital. Echocardiography was performed using Philips Epic CVx 3D, software version7.0.5, ultrasound probes are X5-1, S9-2, X7 S12.

As suggested by AHA guidelines (2, 22), we evaluated the echocardiographic measurements of each coronary artery, right coronary artery (RCA), common trunk (LMCA) and left anterior descending artery (LAD), at onset and at the 2nd, 4th and 8th week follow-ups.

Among the various Z-scores available in the literature, the Boston Z-score was chosen.

We assessed the trend of coronary dilation expressed as Z- score of LMCA, LAD and RCA over an eight weeks observation period in the two groups, HR-S (high-risk patients treated with steroids) and HR-NS (high-risk patients not treated with steroids).

The results were expressed in absolute terms and normalized terms (Z-score divided by baseline value to highlight changes in coronary involvement).

We also analyzed the variation of the AHA risk class in HR-S and HR-NS groups.

Furthermore, to obtain a unique Z-score for each patient we introduced a new calculation methodology:

a. Z-MAX: the maximum value of the scores between the 3 coronary arteries
b. Z-SUM: the sum of the single Z-scores of the 3 coronary arteries

Z-SUM and Z-MAX were obtained by assigning a score according to the following scheme:

• Z-score < 2: score 0
• Z-score ≥ 2 and < 2·5: score 1
• Z-score ≥ 2·5 and < 5: score 2
• Z-score ≥ 5 and < 10: score 3
• Z score ≥ 10: score 4.

The trends of Z-MAX and Z-SUM were analyzed over an eight-week period in the HR-S and HR-NS groups.

### Test used

IBM SPSS Statistics 22.0 was used for data analysis.

Normally distributed continuous variables were presented as mean ± standard deviation (mean ± SD), and non-normally distributed variables were expressed as median (interquartile range, IQR).

The Mann–Whitney *U* test was used for intergroup comparisons.

Fisher's exact test was used to analyze count data.

We set the threshold for statistical significance at a difference of 0.05.

## Results

### Population characteristics data

**Figure 1:**
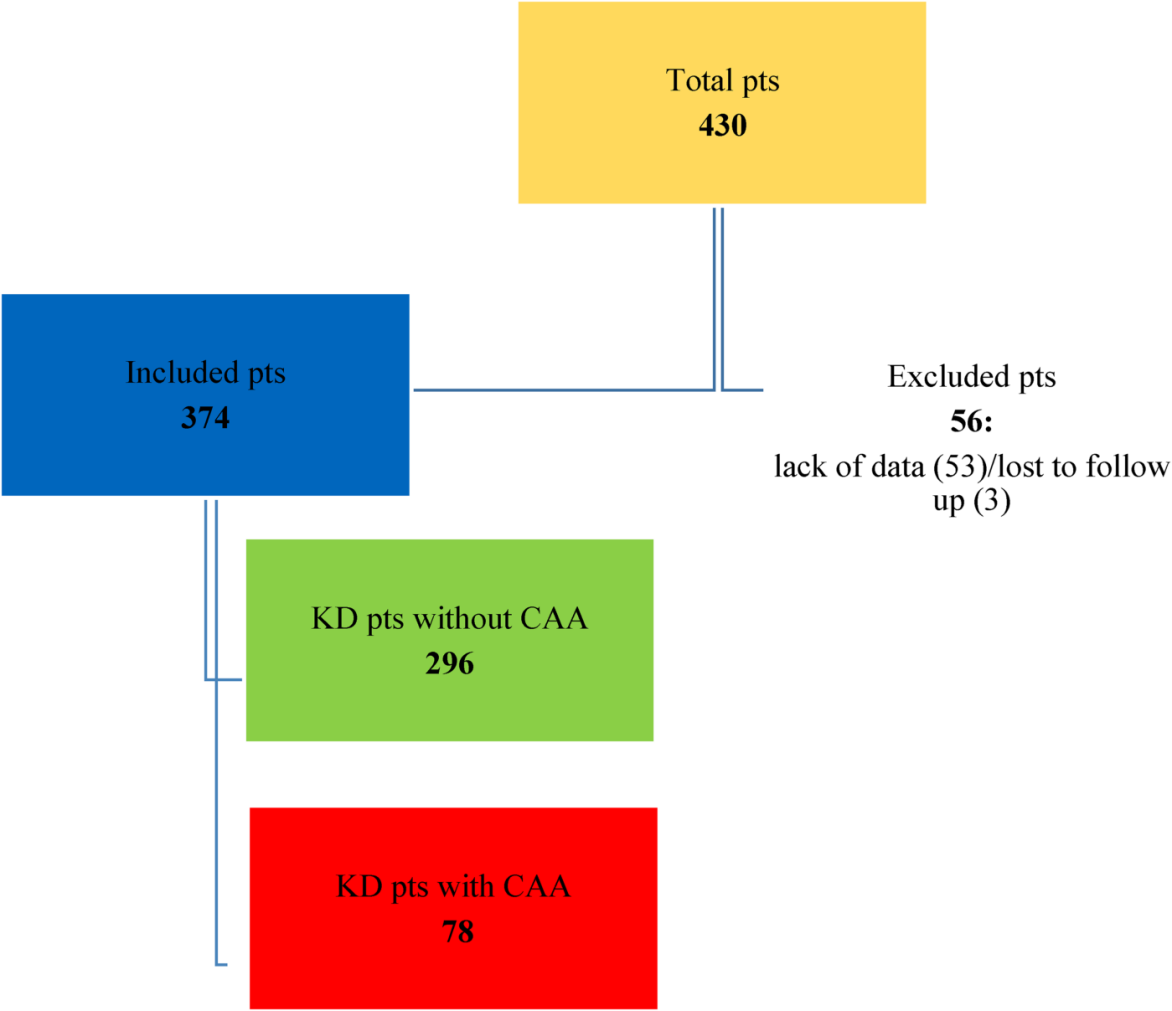
Distribution of total KD population.

**Figure 2:**
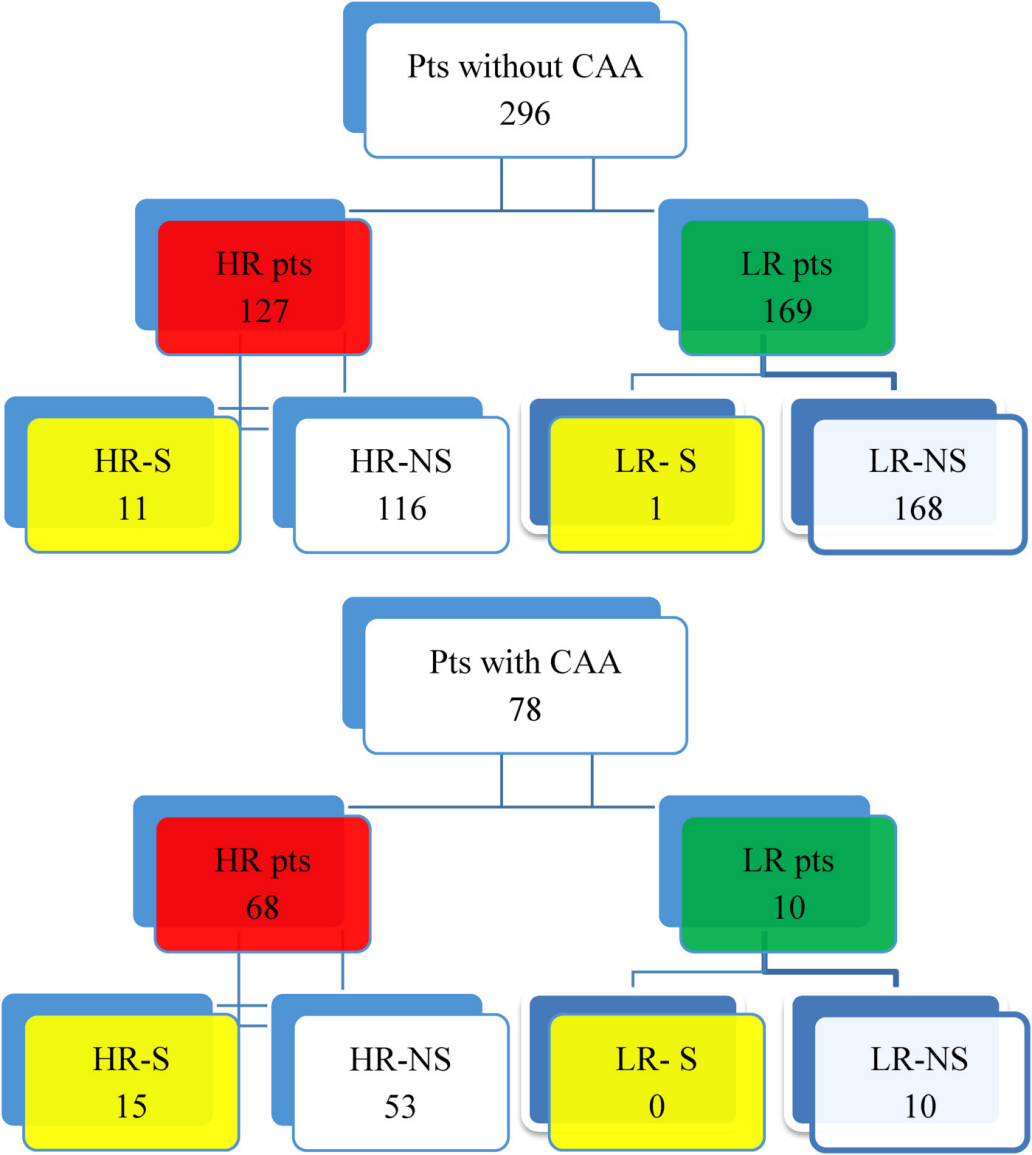
Distribution of KD patients without CAA and affected by CAA. • HR-S: High-risk patients treated with IVIG and steroids as first-line therapy. • HR-NS: High-risk patients who were only treated with IVIG and never with steroids as a first-line therapy. • LR-S: Low-risk patients who were treated with IVIG and steroids as a first-line therapy. • LR-NS: Low-risk patients who only received IVIG and were never treated with steroids as a first-line therapy.

A total of 430 KD patients were admitted to the Bambino Gesù Children's Hospital during the study period. Fifty-six patients were excluded from the study due to incomplete data at the onset of the study, because they were transferred from other hospitals, or because they were lost at the eight-week follow-up. The distribution of the population is shown in Figures 1, 2.

The demographic findings of our population can be summarized as follows:

- Mean age at onset: 19.28 months for KD patients with CAA vs. 35.38 months for those without CAA.
- Patients aged ≤12 months: 53.8% in the CAA group and 17.2% in the non-CAA group, confirming the higher risk of developing CAA in this age group.
- Male: Female ratio = 3:1 among KD patients with CAA (higher than reported in the literature) and 1.5:1 among KD patients without CAA.

Notably, ten LR patients within the CAA group were classified as low risk because their CAA developed after disease onset and following initial treatment with IVIG alone, in accordance with standard risk stratification criteria.

### Data processing

#### Specific Z-score analysis

**Figure 3:**
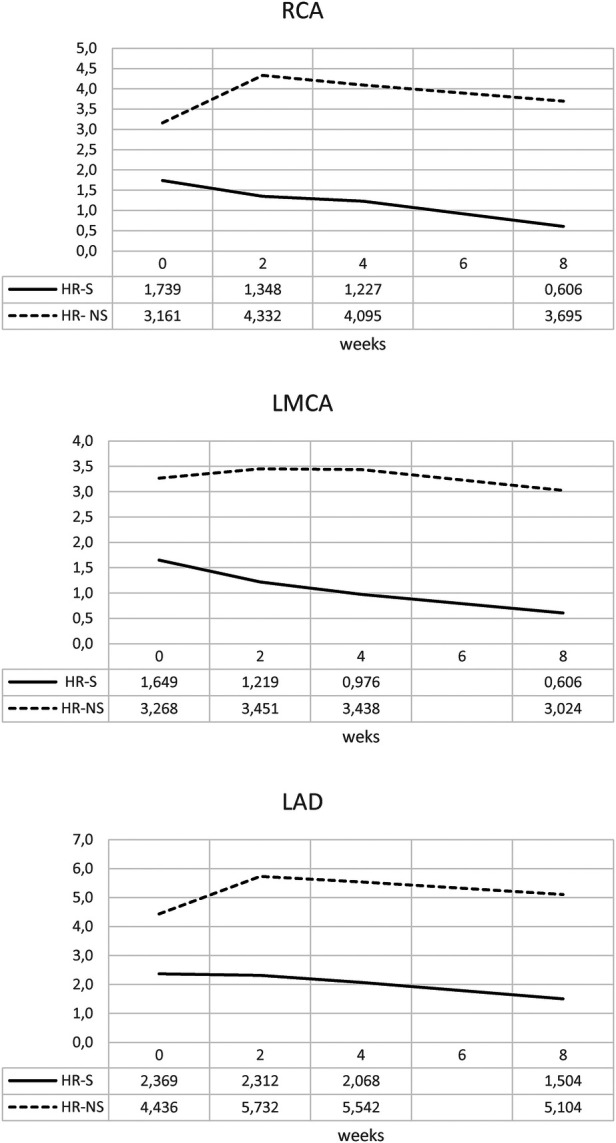
Time trend of mean Z-score of RCA, LMCA and LAD coronary arteries in HR-S and HR-NS group.

The temporal evolution of the Z-score was evaluated in the HR- S population steroids treated compared with the HR-NS population (Figure 3 and Table 1).

**Table 1:** Mean of Z-score of RCA, LMCA and LAD coronary arteries in HR-S and HR-NS group.

GROUP	0 weeks	2 weeks	4 weeks	8 weeks
RCA HR-S	1.739	1.348	1.227	0.606
RCA HR- NS	3.161	4.332	4.095	3.695
LMCA HR-S	1.649	1.219	0.976	0.606
LMCA HR-NS	3.268	3.451	3.438	3.024
LAD HR-S	2.369	2.312	2.068	1.504
LAD HR-NS	4.436	5.732	5.542	5.104

The HR-S group had a more severe initial condition, as indicated by a higher initial Z-score.

By the end of eight weeks, the HR-S group showed a significant decrease in all coronary artery dimensions (RCA decreased by 65%, LMCA decreased by 63%, and LAD decreased by 36.5%), suggesting that immediate steroid treatment was significantly more effective in reducing the Z-score.

In contrast, the HR-NS group recorded an increase in the dimensions of two arteries (the RCA increased by 17%, and the LAD increased by 15%), as well as a slight reduction in the LMCA dimension (7.5%).

**Figure 4:**
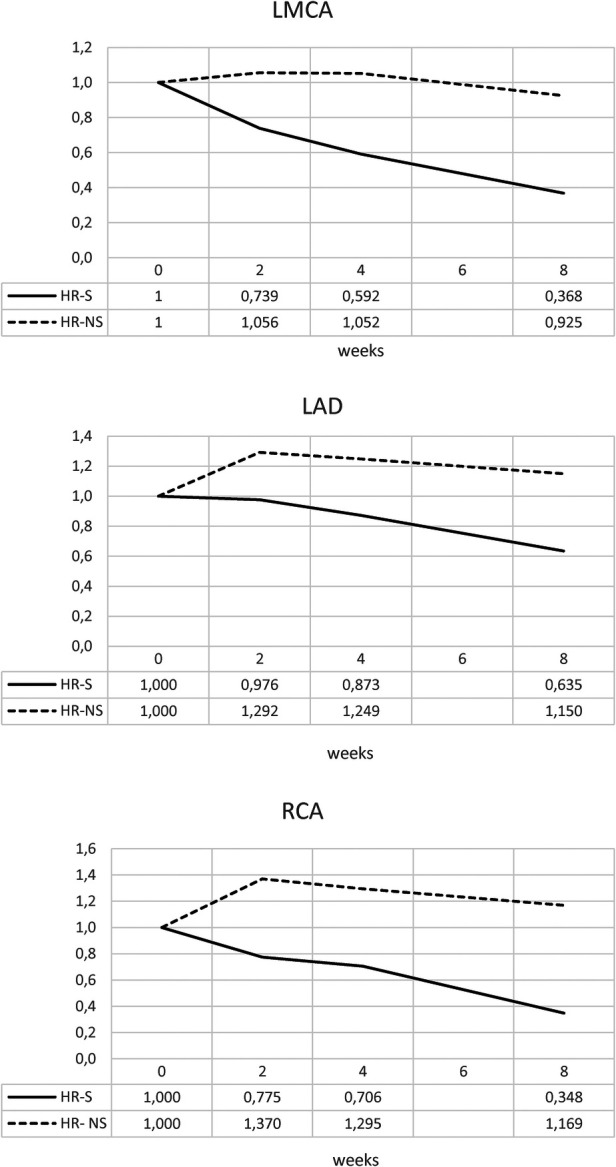
Time trend of normalized Z-score of RCA, LMCA and LAD coronary arteries in HR-S and HR-NS group.

Similar results were obtained for the normalized Z-scores of the RCA, LMCA, and LAD coronary arteries, underlining a dimensional reduction of the CAA in the steroid-treated population, regardless of the initial Z-score value of each group (Figure 4 and Table 2).

**Table 2:** Mean of normalized Z-score of RCA, LMCA and LAD coronary arteries in HR-S and HR-NS group.

GROUP	0 weeks	2 weeks	4 weeks	8 weeks
RCA HR-S	1.0	0.775	0.706	0.348
RCA HR- NS	1.0	1.37	1.295	1.169
LMCA HR-S	1.0	0.739	0.592	0.368
LMCA HR-NS	1.0	1.056	1.052	0.925
LAD HR-S	1.0	0.976	0.873	0.635
LAD HR-NS	1.0	1.292	1.249	1.15

**Figure 5:**
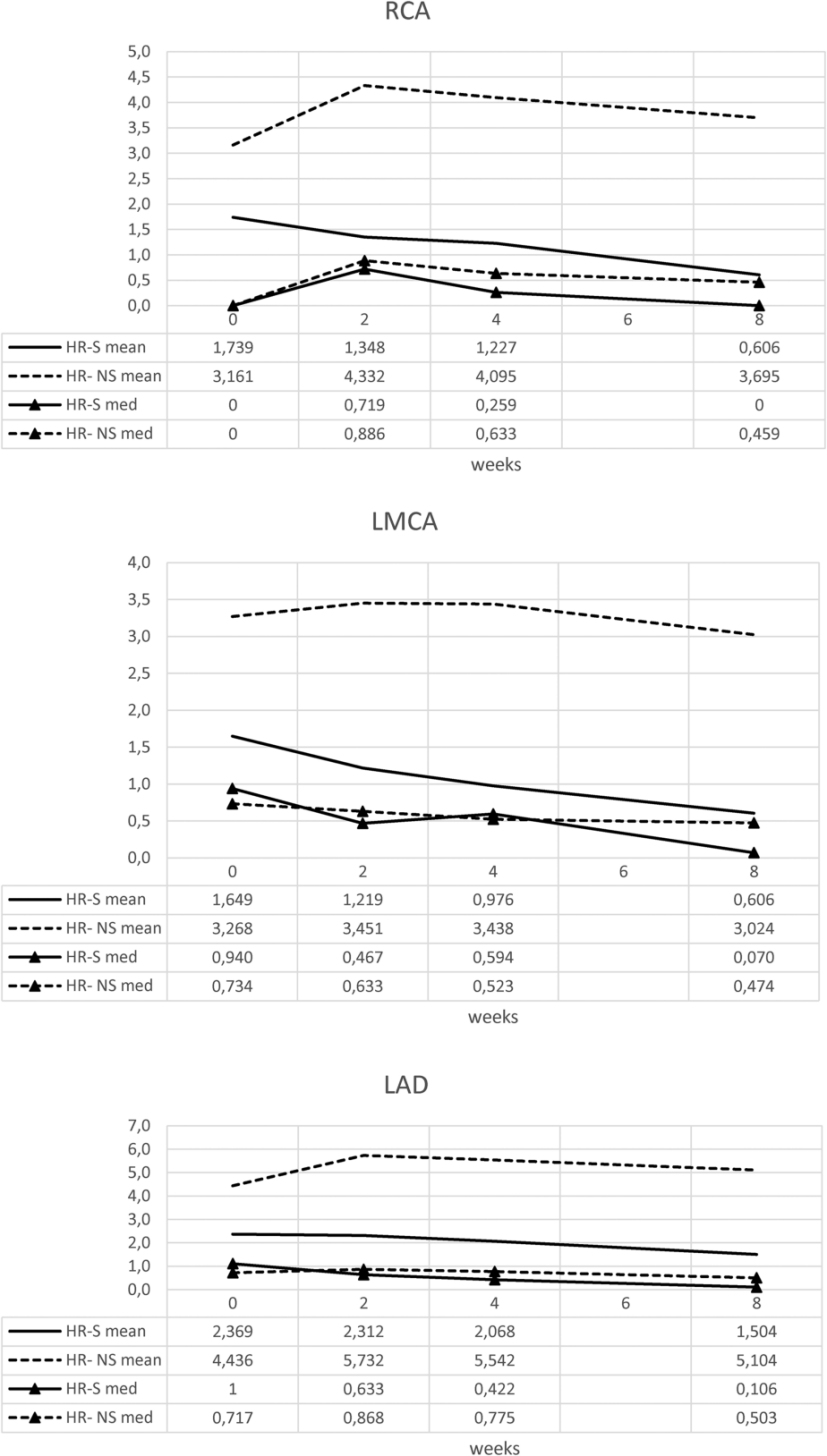
Time trend of mean and median of normalized Z-score of RCA, LMCA and LAD coronary arteries in HR-S and HR-NS group.

The trends of the mean and median for the normalized HR-S and HR-NS groups for all three coronary arteries showed considerable advantages of steroid therapy (Figure 5 and Table 3).

**Table 3:** Median of normalized Z-scores of RCA, LMCA and LAD coronary arteries in HR-S and HR-NS group, with Mann–Whitney *U* test *p*-values for comparisons at 8 weeks.

GROUP	0 weeks	2 weeks	4 weeks	8 weeks	*p*-value
RCA HR-S	0.607	0.719	0.259	0.0	0.046
RCA HR- NS	0.886	0.886	0.633	0.459	
LMCA HR-S	0.94	0.467	0.594	0.07	0.013
LMCA HR-NS	0.734	0.633	0.523	0.474	
LAD HR-S	1.106	0.633	0.422	0.106	0.107
LAD HR-NS	0.717	0.868	0.775	0.503	

**Figure 6:**
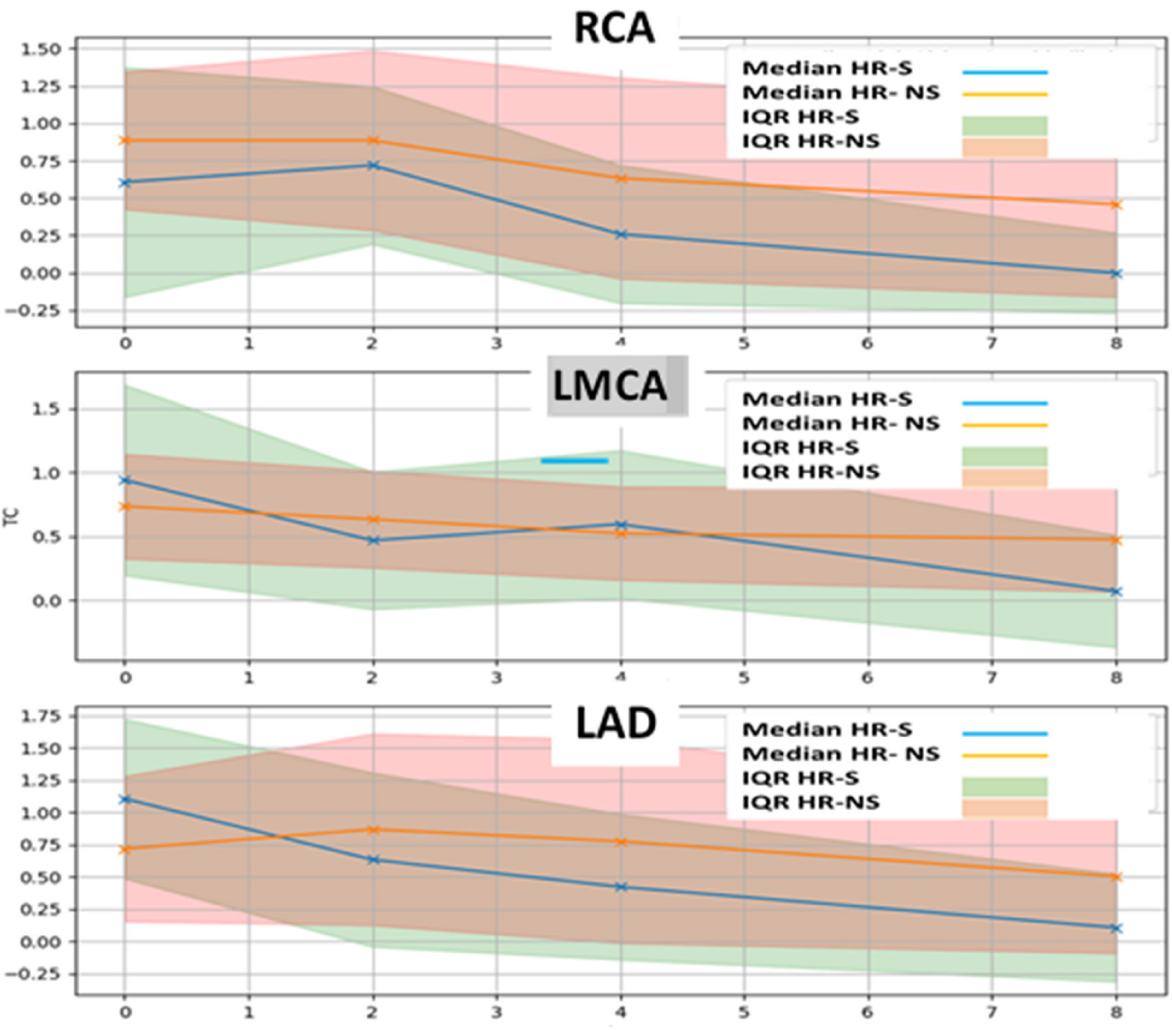
Time trend of median and interquartile of normalized Z-score of RCA, LMCA and LAD coronary arteries in HR-S and HR-NS group.

Furthermore, a strong interquartile range of normalized Z-score overlap between the two groups was evident (Figure 6 and Tables 4, 5).

**Table 4:** The standard deviation of normalized Z-score of RCA, LMCA and LAD coronary arteries in HR-S and HR-NS group.

GROUP	0 weeks	2 weeks	4 weeks	8 weeks
RCA HR-S	1.049	1.065	1.443	1.003
RCA HR- NS	1.016	1.701	1.986	1.963
LMCA HR-S	0.931	0.672	0.963	0.634
LMCA HR-NS	1.041	1.554	1.846	1.827
LAD HR-S	0.862	1.49	1.361	1.293
LAD HR-NS	1.133	1.509	1.668	1.718

**Table 5:** Interquartile range of normalized Z-score of RCA, LMCA and LAD coronary arteries in HR-S and HR-NS group.

GROUP	0 weeks	2 weeks	4 weeks	8 weeks
RCA HR-S	1.537	1.055	0.923	0.538
RCA HR- NS	0.921	1.202	1.344	1.24
LMCA HR-S	1.492	1.075	1.158	0.879
LMCA HR-NS	0.82	0.759	0.731	0.82
LAD HR-S	1.237	1.351	1.129	0.837
LAD HR-NS	1.127	1.485	1.578	1.192

**Table 6:** Time trend of Z-MAX, Z-MAX normalized, Z-SUM and Z-SUM normalized scores in HR-S and HR-NS group, with Mann–Whitney *U* test *p*-values for comparisons at 8 weeks.

SCORE	GROUP	0 weeks	2 weeks	4 weeks	8 weeks	*p*-value
Z-MAX	HR-S	1.12	0.88	0.72	0.44	-
	HR-NS	0.65	0.67	0.62	0.51	-
	all HR	0.706	0.701	0.634	0.505	-
Z-MAX NORM	HR-S	1	0.79	0.64	0.39	0.0056
	HR-NS	1	1.05	0.96	0.80	
	all HR	1	0.992	0.898	0.715	-
Z-SUM	HR-S	1.84	1.32	1.00	0.72	-
	HR-NS	1.33	1.45	1.29	1.08	-
	all HR	1.392	1.433	1.253	1.031	-
Z-SUM NORM	HR-S	1	0.72	0.54	0.39	0.1199
	HR-NS	1	1.09	0.97	0.81	
	all HR	1	1.030	0.900	0.741	-

The Mann–Whitney *U* test shows that the result is statistically significant for RCA and LAD, while not significant for TC in the two treatment groups (Mann–Whitney U *p*-value for RCA: 0.046, for LAD: 0.013, for LMCA: 0.107).

#### Z-MAX e Z-SUM analysis

However, since patients often have multiple coronary dilations, we used Z-MAX and Z-SUM, as previously mentioned, to evaluate the overall trend of patients' coronary circulation.

**Figure 7:**
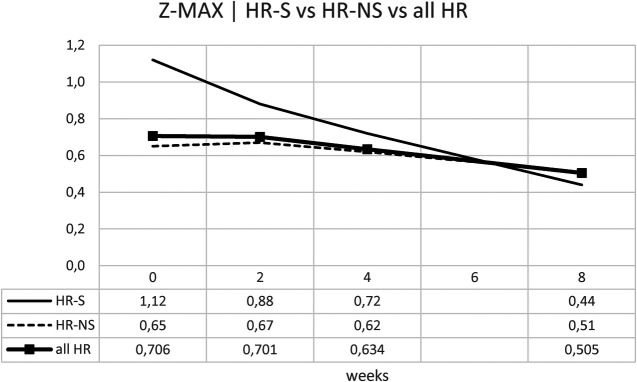
Time trend of the Z-MAX.

We evaluated the temporal evolution of CAA expressed as Z-MAX in the HR-S, HR-NS, and all HR groups (representing the combined HR-S and HR-NS groups). As expected, the HR-S group had a more severe initial condition, as indicated by a higher initial Z-MAX score. However, the HR-S group showed a more pronounced reduction in the Z-MAX curve (from 1.12–0.44), compared to the HR-NS group (from 0.64–0.51). There was a significant improvement in the Z-score at eight weeks (Table 6 and Figure 7). These results suggest that immediate treatment with steroids was markedly more effective in reducing the Z-MAX score in the HR-S population.

**Figure 8:**
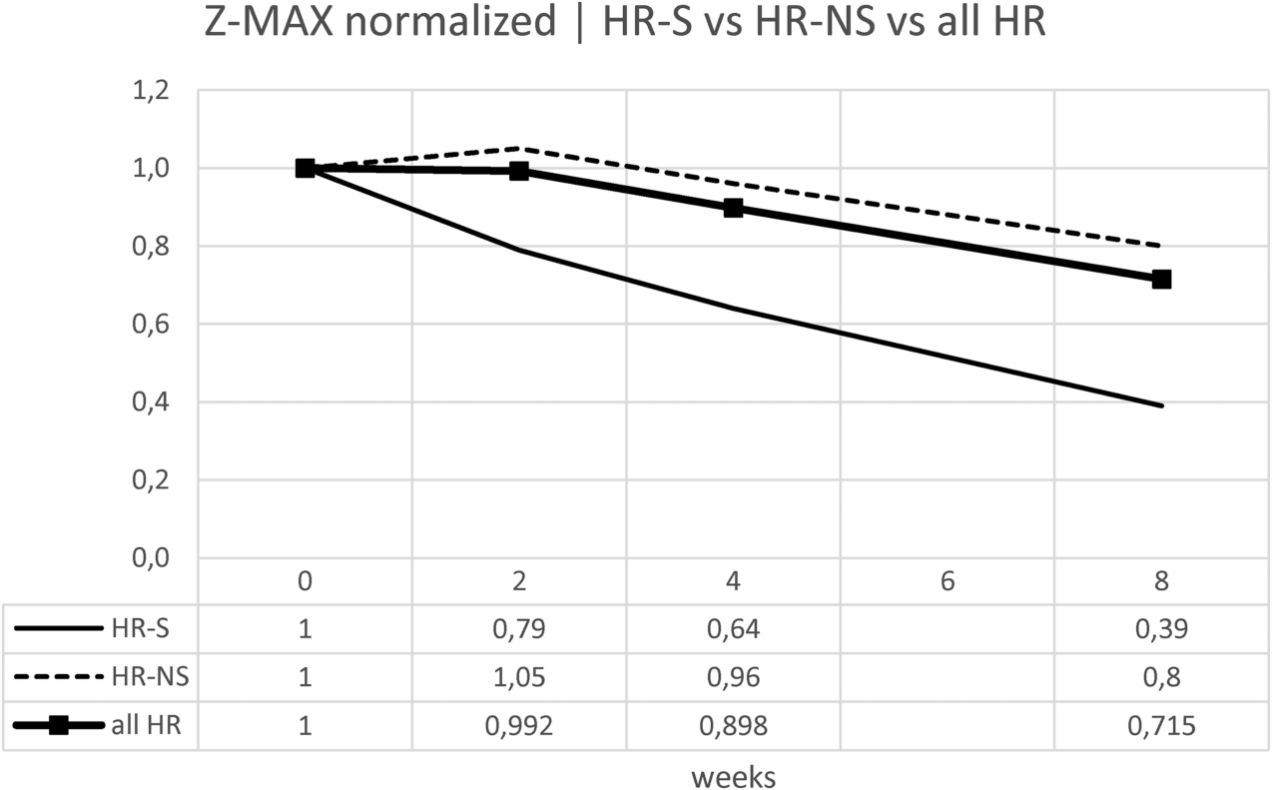
Time trend of the normalized Z-MAX.

Normalizing the previous results, the percentage change is displayed: for the Z-MAX, a variation of 60% was observed in HR-S patients vs. a variation of less than 20% in HR-NS patients (Figure 8 and Table 6).

**Figure 9:**
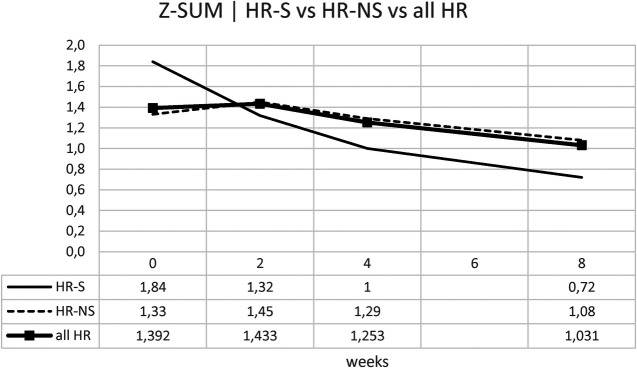
Time trend of the Z-SUM.

Considering the Z-SUM, the benefit of early steroid therapy was observed even more pronounced, showing a significant reduction in Z-SUM at eight weeks after onset in the HR-S population compared to the HR-NS population (Figure 9 and Table 6).

**Figure 10:**
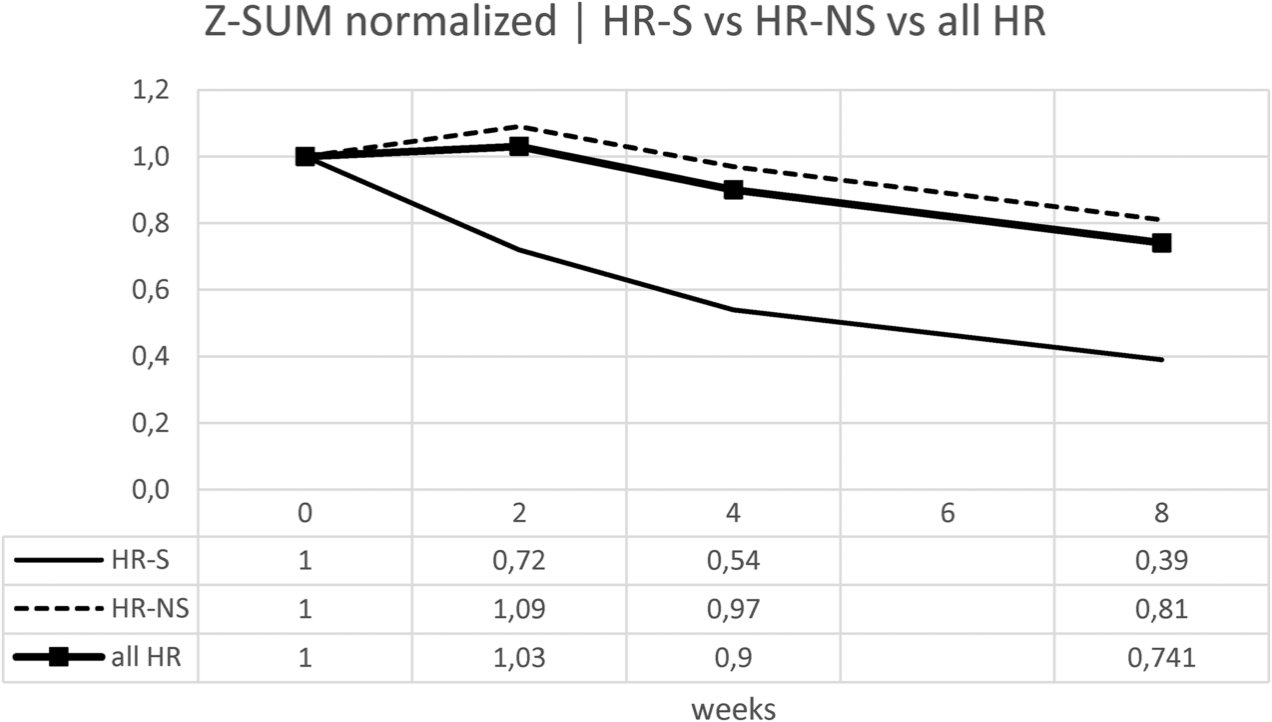
Time trend of the normalized Z-SUM.

Similarly for the Z-SUM, it was noted a variation in HR- S patients of 61% vs. 19% in HR- NS patients (Figure 10 and Table 6).

Trying to consider the change in z-score between onset and 8 weeks of both standardized Z-MAX and standardized Z-SUM in the HR-S and HR-NS groups, using the Mann–Whitney *U* test, we observed a statistically significant difference for the change in Z-MAX HR-S and HR-NS (*p*-value: 0.0056), not for the change in Z-SUM HR-S and HR-NS (*p*-value: 0.1199).

Finally, we compared the AHA coronary risk classes (20) at the eighth-week follow-up between patients treated early with steroids or subjected to conventional treatment: 67% of patients with complicated MK treated early with steroids showed complete regression of CAA, vs. 33% of patients not treated.

Specifically, the odds of having persistent CAA (AHA class III–V) (22) at eight weeks were 0.38 in the steroid-treated group (HR-S), compared to 1.56 in the untreated group (HR-NS). This corresponds to an odds ratio (OR) of 0.24, indicating that patients treated with steroids had a 76% lower odds of having a persistent aneurysm than those not receiving steroids (Table 7). The odds ratio (HR-S vs. HR-NS) was 0.24 (*p*-value = 0.01229).

**Table 7:** Number of transient and persistent CAA cases and corresponding odds of persistence (AHA class III–V) in HR-NS and HR-S groups.

AHA class	HR- NS	Odds	HR- S	Odds
Transient CAA if AHA class II	25		16	
Persistent CAA if AHA class III + IV + V	39	1.56	6	0.38

**Table 8:** Comparison of ORs between individual AHA risk classes (*p*-value = 0.016).

AHA class	HR- NS	HR-S	OR	CI 95%
Class II	2	4	0.25	0.023–2.76
Class III	23	12	3.67	1.37–9.85
Class IV	25	4	39.06	8.78–173.79
Classe V	14	2	49.0	6.03–398.28

Fisher's exact test yielded a *p*-value of 0.01229 (95% CI: 0.08293, 0.69676), confirming a significant relationship between steroid treatment and risk class.

Similar results were obtained by analyzing the individual risk classes in the two treatment groups. A chi-square test yielded a significant *p*-value of 0.016, demonstrating the benefit of steroid use across the risk class distribution (Table 8).

## Discussion

Prevention of the development of CAA constitutes a crucial point in the management of KD patients.

Current guidelines recommend first-line therapy involving the combined use of intravenous immunoglobulin (IVIG) and corticosteroids for patients at high risk for IVIG resistance or coronary aneurysm development (2, 3, 21, 22). Consequently, identifying high-risk (HR) patients has been the focus of extensive research.

We previously described distinct immune profiles and response trajectories in high risk KD patients, suggesting the need for alternative therapeutic strategies (23, 24).

As Burns (25) recall us, the first physician to use steroids for acute KD was Dr Tomisaku Kawasaki himself, who treated 21 patients with various forms of parenteral or oral steroids (1).

He observed that “Clearly intravenous infusion of prednisolone was effective in terminating the fever and improving the general condition, but it is not clear that it helped to shorten the overall course of this syndrome, since we did not see a significant difference when compared with the other treatment groups”. Burns noticed that at that time he was unaware of the potential cardiovascular complications of KD at that time and so his conclusions were based solely on his clinical observation.

Several reports have since supported steroid use as first-line treatment: the Japanese “RAISE” (13) study, and subsequent “post-RAISE” (14), further studies such as those by Inoue et al. (26), Chen et al. (27), Yang et al. (28), Ae et al. (29). In particular, the study by Inoue et al. used prednisolone (2 mg/kg) with IVIG until defervescence. Despite the confounding factor of doses of aspirin and prednisolone that were inconsistent with the recommended European doses, a significant difference in CAA incidence was demonstrated (26). A 2013 meta-analysis by Chen et al. (27) including 16 comparative studies and Yang's 2017 meta-analysis (28) demonstrated that the early addition of steroids to IVIG reduced the risk of CAA. A 2017 Cochrane review examined seven trials with 922 participants. Pooled analysis revealed that corticosteroids reduced the subsequent occurrence of coronary artery abnormalities (OR 0.29, 95% CI 0.18–0.46; 907 participants) without causing serious adverse events or mortality: these evidence was considered moderate for the incidence of CAA due to potential inconsistencies in data geography and patient benefits according to grouping. The authors concluded that subgroup analysis showed some groups that may benefit more than others, but that further randomized controlled trials were required before this could be the basis for clinical action (30, 31).

In a 2020 randomized study, Ae et al. used combined treatment with multiple-dose steroids in a large sample of Japanese KD patients demonstrating a significant reduction in coronary dilatation and resistance (29).

Meanwhile, Eleftheriou et al. have started a randomized, controlled, open-label, endpoint-based, blind-evaluated multicenter study called KD-CAAP, whose results are in processing phase. The study aims to explore the effectiveness of additional corticosteroids in preventing CAA (32).

In 2022, Dionne, Burns, Newburger, and Son conducted a retrospective multicenter study in children with CAA (z-score ≥2.5 and <10 at diagnosis), comparing IVIG monotherapy to combination treatments with corticosteroids or infliximab. Of the 121 children in the study, 25% received corticosteroids and IVIG, 48% received infliximab and IVIG, and 27% received IVIG alone. Both corticosteroid and infliximab combination therapies were independently associated with less coronary progression (coefficients: −1.31 and −1.07, respectively), compared to IVIG alone (33).

Iio et al. concluded that even predicted IVIG responders with CAA risk factors in the Japanese population might benefit from intensified first-line therapy using corticosteroids, cyclosporine, or infliximab (34).

In 2024, Miura et al. published the results of a large, prospective, multicenter observational study involving 28 Japanese hospitals from 2016–2020. Hospitals independently opted to add corticosteroid regimens to IVIG for patients with a Kobayashi score ≥5 and total bilirubin ≥1.0 mg/dl. Although adjunctive corticosteroids improved treatment response and inflammation control, the study did not find additional benefits from adding methylprednisolone IV to prednisolone. Coronary outcomes in patients treated with IVIG alone were comparable, though they required more rescue therapy (35).

Yang et al. (15). by their findings provided valuable insights into the potential mechanisms underlying the therapeutic effects of IVIG plus methylprednisolone by performing single-cell analysis on 14 peripheral blood mononuclear cell samples obtained from seven KD patients who received either IVIG monotherapy or IVIG plus methylprednisolone therapy. They demonstrated that both IVIG monotherapy and IVIG plus methylprednisolone therapy can increase lymphocyte counts (e.g., CD4+ T, CD8+ T, and gdT cells) to address lymphopenia, decrease monocyte counts and repress the expression of S100A12, NLRP3, and genes associated with immune cell migration in monocytes. Futhermore, IVIG combined with methylprednisolone downregulates more monocyte-driven inflammatory pathways than IVIG alone. Additionally, this combination uniquely enhances NK cell cytotoxicity by modulating receptor homeostasis, while significantly upregulating interferon-related genes in CD4+ T cells, CD8+ T cells, and B cells, particularly type I interferons. Therefore, these findings lead us to conclude conclude that the combination of IVIG and methylprednisolone attenuates monocyte-driven inflammation and improves NK cell cytotoxicity.

Our study used Z-MAX and Z-SUM calculation methodology and normalized analyses to show CAA evolution, highlighting a clearer reduction in CAA size in high-risk (HR) patients treated with steroids (Figures 7–10).

At the eight-week follow-up, considering the patient's risk class, 67% of complicated KD patients who were treated early with steroids showed complete CAA regression (risk class II), compared to only 33% of untreated complicated KD patients. The comparison of odds ratios (OR) between transient dilation (AHA risk class II) and persistent dilation (AHA classes III-V) and between individual AHA risk classes demonstrates the clear advantage of steroid therapy for high-risk patients: a 25% reduction in the risk of persistent dilations.

Our findings are consistent with recent literature. The 2017 (2) and 2024 (22) AHA guidelines recommended corticosteroids as the initial treatment for high-risk patients before vascular remodeling occurs, as suggested by a Cochrane meta-analysis (30, 31). A 2021 study by Friedman (36) highlighted that first-line corticosteroids were associated with a greater probability of coronary dilatation regression if present at diagnosis, as well as reduced IVIG resistance and inflammatory indices.

In our study, after eight weeks, the HR-NS group showed a significant decrease in coronary artery dimensions, as measured by Z-score (RCA decreased by 65%, LMCA by 63%, and LAD by 36.5%). This suggests that immediate steroid treatment is significantly more effective in reducing Z-score.

In contrast, the HR-NS group recorded an increase in two artery dimensions (RCA increased by 17%, and the LAD increased by 15%), as well as a slight reduction in the LMCA dimension (7.5%).

Similar results were obtained for the normalized Z-scores of the RCA, LMCA, and LAD coronary arteries, underlining a dimensional reduction of CAA in the steroid-treated population, regardless of the initial Z-score value of each group.

In contrast, the HR- NS group showed an increase in the dimension of two arteries (RCA increased by 17% and LAD increased by 15%) and only a slight reduction of LMCA (7.5%).

Similar results were obtained for the normalized Z-score of RCA, LMCA and LAD coronary arteries, indicating a dimensional reduction of CAA in the steroid treated group, regardless of the initial Z-score value of each group.

Although our study population is small, the results are statistically significant and could contribute to the development of novel targeted therapies for KD, given that our study is retrospective and single-center.

However, our study has limitations. It is a retrospective study conducted at a single center with a limited number of patients; therefore, it was not possible to draw conclusive data on the effectiveness of steroid treatment. Larger, multicenter, randomized controlled trials (RCTs) should be conducted to reach definitive conclusions.

Emerging therapies, such as IL-1 antagonists (e.g., anakinra), have shown promise, though standardized dosing and treatment durations remain undefined and cost remains a concern (37, 38).

In summary, our data, in agreement with the literature, highlight the effectiveness and safety of first-line combined therapy with IVIG and steroids in reducing CAA in high-risk KD patients.

The literature has widely demonstrated that steroid treatment reduces inflammatory indices and the duration of symptoms, consequently reducing hospitalization. This has clear advantages from both a health and economic point of view, as well as for the child's overall health.

## Conclusions and future prospects

Future prospective studies conducted on larger populations may allow for the development of more specific and sensitive risk scores by introducing more stringent cut-offs or different biochemical markers. These studies may also identify one or more risk factors that have a greater negative influence on outcomes. Additionally, future research should address strengthening the evidence on the early use of steroid therapy for all patients, regardless of risk class, to eliminate the risk of developing CAA.

Ultimately, identifying the most cost-effective and clinically beneficial treatment strategies will be essential for improving KD outcomes.

## Data Availability

The raw data supporting the conclusions of this article will be made available by the authors, without undue reservation.

## References

[1] Kawasaki T. Acute febrile mucocutaneous syndrome with lymphoid involvement with specific desquamation of the fingers and toes in children. Arerugi. (1967) 16(3):178–222. 6062087

[2] McCrindle BW Rowley AH Newburger JW Burns JC Bolger AF Gewitz M Diagnosis, treatment, and long-term management of Kawasaki disease: a scientific statement for health professionals from the American heart association. Circulation. (2017) 135(17):e927–99. 10.1161/CIR.0000000000000484 28356445

[3] Marchesi A Rigante D Cimaz R Ravelli A Tarissi de Jacobis I Rimini A Revised recommendations of the Italian society of pediatrics about the general management of Kawasaki disease. Ital J Pediatr. (2021) 47(1):16. 10.1186/s13052-021-00962-4 33494789 PMC7830049

[4] Dionne A Hanna B Trinh Tan F Desjardins L Lapierre C Déry J Importance of anatomical dominance in the evaluation of coronary dilatation in Kawasaki disease. Cardiol Young. (2017) 27(5):877–83. 10.1017/S1047951116001426 27640521

[5] Durongpisitkul K Gururaj VJ Park MJ Martin CF. The prevention of coronary artery aneurysm in Kawasaki disease: a meta-analysis on the efficacy of aspirin and immunoglobulin treatment. Pediatrics. (1995) 96(6):1057–61. 7491221

[6] Chen S Dong Y Kiuchi MG Wang J Li R Ling Z Coronary artery complication in Kawasaki disease and the importance of early intervention: a systematic review and meta-analysis. JAMA Pediatr. (2016) 170(12):1156–63. 10.1001/jamapediatrics.2016.2055 27749951

[7] Tremoulet AH Best BM Song S Wang S Corinaldesi E Eichenfield JR Resistance to intravenous immunoglobulin in children with Kawasaki disease. J Pediatr. (2008) 153(1):117–21. 10.1016/j.jpeds.2007.12.021 18571548 PMC2526555

[8] Davies S Sutton N Blackstock S Gormley S Hoggart CJ Levin M Predicting IVIG resistance in UK Kawasaki disease. Arch Dis Child. (2015) 100(4):366–8. 10.1136/archdischild-2014-307397 25670405

[9] Lyskina G Bockeria O Shirinsky O Torbyak A Leontieva A Gagarina N Cardiovascular outcomes following Kawasaki disease in Moscow, Russia: a single center experience. Glob Cardiol Sci Pract. (2018) 2017:e201723. 10.21542/gcsp.2017.23 PMC5856972 29564344

[10] Kobayashi T Inoue Y Takeuchi K Okada Y Tamura K Tomomasa T Prediction of intravenous immunoglobulin unresponsiveness in patients with Kawasaki disease. Circulation. (2006) 113(22):2606–12. 10.1161/CIRCULATIONAHA.105.592865 16735679

[11] Kato H Koike S Yokoyama T. Kawasaki disease: effect of treatment on coronary artery involvement. Pediatrics. (1979) 63(2):175–9. 10.1542/peds.63.2.175 440805

[12] Newburger JW Sleeper LA McCrindle BW Minich LL Gersony W Vetter VL Randomized trial of pulsed corticosteroid therapy for primary treatment of Kawasaki disease. N Engl J Med. (2007) 356(7):663–75. 10.1056/NEJMoa061235 17301297

[13] Kobayashi T Saji T Otani T Takeuchi K Nakamura T Arakawa H Efficacy of immunoglobulin plus prednisolone for prevention of coronary artery abnormalities in severe Kawasaki disease (RAISE study): a randomised, open-label, blinded-endpoints trial. Lancet. (2012) 379(9826):1613–20. 10.1016/S0140-6736(11)61930-2 22405251

[14] Miyata K Kaneko T Morikawa Y Sakakibara H Matsushima T Misawa M Efficacy and safety of intravenous immunoglobulin plus prednisolone therapy in patients with Kawasaki disease (post RAISE): a multicentre, prospective cohort study. Lancet Child Adolesc Health. (2018) 2(12):855–62. 10.1016/S2352-4642(18)30293-1 30337183

[15] Yang M Chen Y Feng C Zhang M Wang H Zheng Y Single-cell RNA sequencing uncovers molecular mechanisms of intravenous immunoglobulin plus methylprednisolone in Kawasaki disease: attenuated monocyte-driven inflammation and improved NK cell cytotoxicity. Front Immunol. (2024) 15:1455925. 10.3389/fimmu.2024.1455925 39524437 PMC11543420

[16] de Graeff N Groot N Ozen S Eleftheriou D Avcin T Bader-Meunier B European consensus-based recommendations for the diagnosis and treatment of Kawasaki disease–the SHARE initiative. Rheumatology. (2019) 58(4):672–82. 10.1093/rheumatology/key344 30535127

[17] Athappan G Gale S Ponniah T. Corticosteroid therapy for primary treatment of Kawasaki disease—weight of evidence: a meta-analysis and systematic review of the literature. Cardiovasc J Afr. (2009) 20(4):233–6. 19701534 PMC3734755

[18] Fabi M Andreozzi L Corinaldesi E Bodnar T Lami F Cicero C Inability of Asian risk scoring systems to predict intravenous immunoglobulin resistance and coronary lesions in Kawasaki disease in an Italian cohort. Eur J Pediatr. (2019) 178(3):315–22. 10.1007/s00431-018-3297-5 30499051

[19] Eleftheriou D Levin M Shingadia D Tulloh R Klein N Brogan P. Management of Kawasaki disease. Arch Dis Child. (2014) 99(1):74–83. 10.1136/archdischild-2012-302841 24162006 PMC3888612

[20] Son MBF Gauvreau K Tremoulet AH Lo M Baker AL de Ferranti S Risk model development and validation for prediction of coronary artery aneurysms in Kawasaki disease in a North American population. J Am Heart Assoc. (2019) 8(11):e011319. 10.1161/JAHA.118.011319 31130036 PMC6585355

[21] Gorelik M Chung SA Ardalan K Binstadt BA Friedman K Hayward K 2021 American college of rheumatology/vasculitis foundation guideline for the management of Kawasaki disease. Arthritis Care Res. (2022) 74(4):538–48. 10.1002/acr.24838 PMC12323682 35257507

[22] Jone PN Tremoulet A Choueiter N Dominguez SR Harahsheh AS Mitani Y Update on diagnosis and management of Kawasaki disease: a scientific statement from the American heart association. Circulation. (2024) 150(23):e481–500. 10.1161/CIR.000000000000129 39534969

[23] Cotugno N Olivieri G Pascucci GR Amodio D Morrocchi E Pighi C Multi-modal immune dynamics of pre-COVID-19 Kawasaki disease following intravenous immunoglobulin. Clin Immunol. (2024) 267:110349. 10.1016/j.clim.2024.110349 39186994

[24] Consiglio CR Cotugno N Sardh F Pou C Amodio D Rodriguez L The immunology of multisystem inflammatory syndrome in children with COVID-19. Cell. (2020) 183(4):968–981.e7. 10.1016/j.cell.2020.09.016 32966765 PMC7474869

[25] Burns JC. Revisiting once again steroids for the treatment of acute Kawasaki disease. J Am Heart Assoc. (2020) 9(17):e018300. 10.1161/JAHA.120.018300 32811264 PMC7660753

[26] Inoue Y Okada Y Shinohara M Kobayashi T Kobayashi T Tomomasa T A multicenter prospective randomized trial of corticosteroids in primary therapy for Kawasaki disease: clinical course and coronary artery outcome. J Pediatr. (2006) 149(3):336–341.e1. 10.1016/j.jpeds.2006.05.025 16939743

[27] Chen S Dong Y Yin Y Krucoff MW. Intravenous immunoglobulin plus corticosteroid to prevent coronary artery abnormalities in Kawasaki disease: a meta-analysis. Heart. (2013) 99(2):76–82. 10.1136/heartjnl-2012-302126 22869678

[28] Yang T-J Lin M-T Lu C-Y Chen J-M Lee P-I Huang L-M The prevention of coronary arterial abnormalities in Kawasaki disease: a meta-analysis of the corticosteroid effectiveness. J Microbiol Immunol Infect. (2018) 51(3):321–31. 10.1016/j.jmii.2017.08.012 28927685

[29] Ae R Abrams JY Maddox RA Schonberger LB Nakamura Y Kuwabara M Corticosteroids added to initial intravenous immunoglobulin treatment for the prevention of coronary artery abnormalities in high-risk patients with Kawasaki disease. J Am Heart Assoc. (2020) 9(17):e015308. 10.1161/JAHA.119.015308 32811256 PMC7660775

[30] Wardle AJ Connolly GM Seager MJ Tulloh RM. Corticosteroids for the treatment of Kawasaki disease in children. Cochrane Database Syst Rev. (2017) 2017(1):CD011188. 10.1002/14651858.CD011188.pub2 PMC6464937 28129459

[31] Green J Wardle AJ Tulloh RM. Corticosteroids for the treatment of Kawasaki disease in children. Cochrane Database Syst Rev. (2022) 2022(5):CD011188. 10.1002/14651858.CD011188.pub3 PMC9139689 35622534

[32] Eleftheriou D Moraes YC Purvis C Pursell M Morillas MM Kahn R Multi-centre, randomised, open-label, blinded endpoint assessed, trial of corticosteroids plus intravenous immunoglobulin (IVIG) and aspirin, versus IVIG and aspirin for prevention of coronary artery aneurysms (CAA) in Kawasaki disease (KD): the KD CAA prevention (KD-CAAP) trial protocol. Trials. (2023) 24(1):60. 10.1186/s13063-022-07051-9 36703139 PMC9879235

[33] Dionne A Burns JC Dahdah N Tremoulet AH Gauvreau K de Ferranti SD Treatment intensification in patients with Kawasaki disease and coronary aneurysm at diagnosis. Pediatrics. (2019) 143(6):e20183341. 10.1542/peds.2018-3341 31048414

[34] Iio K Morikawa Y Miyata K Kaneko T Misawa M Yamagishi H Risk factors of coronary artery aneurysms in Kawasaki disease with a low risk of intravenous immunoglobulin resistance: an analysis of post RAISE. J Pediatr. (2022) 240:158–163.e4. 10.1016/j.jpeds.2021.08.065 34461064

[35] Miura M Miyata K Kaneko T Akahoshi S Morikawa Y Matsushima T Methylprednisolone pulse and prednisolone for intensification of primary treatment in Kawasaki disease patients at high risk of treatment resistance: a multicenter prospective cohort study. Eur J Pediatr. (2024) 183(10):4265–74. 10.1007/s00431-024-05689-y 39048743

[36] Friedman KG Gauvreau K Baker A Son MB Sundel R Dionne A Primary adjunctive corticosteroid therapy is associated with improved outcomes for patients with Kawasaki disease with coronary artery aneurysms at diagnosis. Archs Dis Child. (2021) 106(3):247–52. 10.1136/archdischild-2020-319810 32943389

[37] Lee Y Schulte DJ Shimada K Chen S Crother TR Chiba N Interleukin-1β is crucial for the induction of coronary artery inflammation in a mouse model of Kawasaki disease. Circulation. (2012) 125(12):1542–50. 10.1161/CIRCULATIONAHA.111.072769 22361326 PMC3337219

[38] Gorelik M Lee Y Abe M Andrews T Davis L Patterson J IL-1 receptor antagonist, anakinra, prevents myocardial dysfunction in a mouse model of Kawasaki disease vasculitis and myocarditis. Clin Exp Immunol. (2019) 198(1):101–10. 10.1111/CEI.13314 31099056 PMC6718290

